# The rate and predictors of recompensation in patients with decompensated cirrhosis due to metabolic dysfunction–associated liver disease (MASLD)

**DOI:** 10.1097/HC9.0000000000000919

**Published:** 2026-03-11

**Authors:** Alba Jiménez-Masip, M. Teresa Broquetas, Sergio Muñoz-Martinez, Isabel Serra-Matamala, Diego Rojo, Anna Sòria, Cautar El Maimouni, Laura Pagès, Mònica Pons, José A. Carrión, Pere Ginès, Joan Genescà, Isabel Graupera, Juan M. Pericàs

**Affiliations:** 1 Liver Unit, Digestive Diseases Division, Vall d’Hebron University Hospital, Barcelona, Spain; 2 Vall d’Hebron Institut de Recerca (VHIR), Vall d’Hebron Barcelona Campus Hospitalari, Barcelona, Spain; 3 Faculty of Medicine Universitat Autònoma de Barcelona, Department of Gastroenterology, Barcelona, Spain; 4 Spanish Centers for Biomedical Research in Liver and Digestive Diseases, (CIBERehd), Madrid, Spain; 5 Secció d’Hepatologia, Servei de Digestiu, Hospital del Mar, Barcelona, Spain; 6 Institut Hospital del Mar d’Investigacions Mèdiques (IMIM), Barcelona, Spain; 7 Hepatology Section, Digestive Diseases Department, Hospital Universitari Josep Trueta, Girona, Spain; 8 Liver Unit, Hospital Clínic, Barcelona, Spain; 9 Facultat de Medicina i Ciències de la Salut, Universitat de Barcelona, Barcelona, Spain; 10 Fundació de Recerca Clínic Barcelona-Institut d’Investigacions Biomèdiques August Pi i Sunyer (FRCB-IDIBAPS), Barcelona, Spain

**Keywords:** cirrhosis, liver decompensation, MASLD, metabolic dysfunction–associated steatotic liver disease, NAFLD, outcomes, recompensation

## Abstract

**Background:**

Whether patients with metabolic dysfunction–associated steatotic liver disease (MASLD) cirrhosis can achieve recompensation is still a subject of debate. This study aimed to evaluate the incidence and factors associated with recompensation in patients with decompensated MASLD cirrhosis.

**Methods:**

Cohort study across 4 university hospitals in Barcelona (Spain), enrolling individuals with MASLD cirrhosis at their initial decompensating event. Liver recompensation (Baveno VII) was defined as the absence of clinical decompensation after discontinuation of specific treatment, along with sustained improvement in liver function. Competing-risk regression models were used to identify predictors of recompensation.

**Results:**

Among 124 patients [mean age: 69 years (IQR 62–73), 53% males], 59% had obesity, 74% type 2 diabetes, 66% arterial hypertension, and 47% dyslipidemia. Most patients were Child–Pugh B (61%) with a median MELD-Na score of 11 (IQR 10–16). After a median follow-up of 2.1 years (IQR 0.97–4.53), the 2-year cumulative incidence of recompensation was 24%. Factors associated with recompensation included MELD-Na (aSHR 0.891 [95% CI 0.833–0.953]; *p*=0.001), albumin (aSHR 1.894 [95% CI 1.008–3.297]; *p*=0.024, ascites (aSHR 0.475, [95% CI 0.268–0.841]; *p*=0.011), and multiple decompensation (aSHR 0.151 [94% CI 0.033–0.698]; *p*=0.015) as a first decompensation event. Although a substantial proportion of patients initially achieved recompensation, this was frequently transient, and its apparent survival benefit did not persist after adjustment for liver function and accounting for time-dependence.

**Conclusions:**

Liver recompensation in MASLD cirrhosis occurs in 24% of patients within 2 years after first decompensation and is mainly dependent on basal liver function. However, frequent further decompensation limits its prognostic impact on survival.

## INTRODUCTION

Metabolic dysfunction–associated steatotic liver disease (MASLD), formerly known as nonalcoholic fatty liver disease,^1^ is a rapidly increasing form of chronic liver disease, closely linked to the global prevalence of metabolic syndrome and its key components such as obesity and type 2 diabetes mellitus (T2D).^2^ As a consequence, the number of patients with end-stage forms of MASLD such as cirrhosis and hepatocellular carcinoma (HCC) are also increasing,^3^ which has led to a scenario where MASLD is progressively becoming a leading cause of liver decompensation and transplant.^4^,^5^

Nonetheless, the pathophysiological and clinical natural history of MASLD cirrhosis remains relatively unknown,^5^ with few cohort studies available that provide granular data on the disease trajectory once patients are decompensated.^6^,^7^,^8^ However, it is evident that as the disease progresses, liver-related events increasingly predominate over cardiovascular complications, particularly after the first episodes of decompensation.^4^,^5^,^6^

Current treatment focuses on managing metabolic comorbidities, as there are no approved etiological therapies for MASLD-related cirrhosis. One of the major unresolved questions is how addressing these metabolic comorbidities impacts prognosis once cirrhosis reaches decompensation.

It is well-known that improvements in the various components of metabolic syndrome, such as weight loss, enhanced control of glycosylated hemoglobin, serum cholesterol and triglycerides, and arterial hypertension, as well as regular physical exercise, are associated with significant reductions in steatosis, steatohepatitis, and fibrosis.^9^ However, the evidence is less conclusive for patients with cirrhosis.^5^ Moreover, a substantial proportion of patients fail to maintain such optimal metabolic control in the mid-term.^9^

In particular, the question of whether MASLD cirrhosis can recompense has not been answered. The definition and clinical characteristics of recompensated cirrhosis are currently a topic of intense debate.^10^ According to the 2022 Baveno VII consensus criteria, to document the occurrence of recompensated cirrhosis, 3 conditions must be met: (1) correction of the underlying cause; (2) resolution of complications that define a decompensated state; and (3) improvements in liver function tests.^11^

Although there are currently no approved treatments that directly target the underlying cause of MASLD-related cirrhosis, some individuals in clinical practice exhibit resolution of portal hypertension complications and improvements in liver function tests. However, according to the Baveno criteria, these individuals would not meet the formal definition of recompensation. Therefore, in this study, we aimed to assess the likelihood of recompensation in individuals with MASLD cirrhosis, to describe its incidence and drivers, and to evaluate the impact of clinical recompensation on mortality, in a multicenter cohort of patients with decompensated MASLD cirrhosis.

## METHODS

### Setting and participants

This is a retrospective, multicenter study involving 4 university hospitals in Barcelona (Spain), incorporating patients included in a regional registry conducted in the Catalonia region.

This registry included all patients diagnosed with compensated cirrhosis due to MASLD from January 2009 to December 2018. Patients who developed any episode of decompensation during follow-up, from the time of registry enrollment through January 2024, were retrospectively identified and included in the analysis.

Demographic and clinical characteristics, along with laboratory tests, radiological, endoscopic, and histological data, were recorded at the initial decompensating event, which was considered the baseline date. Patients were followed prospectively until death, liver transplantation (LT), or the last recorded follow-up. Anthropometric and metabolic data, clinical status (clinically decompensated: yes or no), as well as the use of concomitant treatment, including non-selective beta-blockers (NSBBs), diuretics, laxatives, rifaximin, along with the respective doses, were recorded every 6 months.

Patients with cirrhosis due to concomitant etiologies other than MASLD were excluded to ensure that the study focused specifically on MASLD cirrhosis. In the case of alcohol consumption, patients exceeding 20 g/day for females or 30 g/day for males at the time of inclusion in the registry were excluded from the study, as this could obscure the distinction between MASLD and the spectrum of alcohol-associated diseases, including both metabolic and alcohol-associated liver disease (MetALD) and alcohol-related liver disease (ALD). Data on alcohol consumption were recorded based on patients’ self-reports. Additional exclusion criteria included clinically compensated patients and those diagnosed with HCC before another decompensation event.

### Variables/definitions

Diagnosis of MASLD cirrhosis was established in patients with obesity, T2D, or metabolic syndrome with no other attributable cause of liver disease and any of the following: (1) liver biopsy with ≥5% steatosis and/or steatohepatitis and a nonalcoholic steatohepatitis Clinical Research Network score of 4 (F4) or cryptogenic cirrhosis; (2) imaging or endoscopic signs of portal hypertension; (3) presence of steatosis by imaging techniques and liver stiffness measurement (LSM) ≥15 kPa.^11^ Imaging signs of portal hypertension included the presence of enlarged paraumbilical veins, portal-systemic collateral pathways, splenomegaly (>13 cm), dilated portal vein (>13 mm), or biphasic, reverse, or reduced flow (<10 cm/s) in the portal vein. Endoscopic signs of portal hypertension were the presence of gastroesophageal varices or portal hypertensive gastropathy. In some patients, a hepatic venous pressure measurement (HVPG) was performed, and an HVPG value ≥10 mm Hg was indicative of clinically significant portal hypertension. Non-invasive assessment of liver stiffness using transient elastography (FibroScan, Echosens, France) was also performed in accordance with established technical recommendations. These included obtaining more than 10 valid measurements, achieving an interquartile range (IQR) of <30%, and ensuring that patients fasted for at least 3 hours before the procedure to optimize accuracy and reliability.

Acute decompensation was defined as the clinically evident new onset ascites, grade 2 or more hepatic encephalopathy per West-Haven classification, and portal hypertension-related bleeding (PHB) defined as gastrointestinal bleeding attributable to gastroesophageal varices and confirmed endoscopically.^12^

### Endpoints

The primary endpoint was recompensation based on Baveno VII criteria and defined as sustained absence of clinical decompensation (at least for 12 months in PHB) without specific treatment (NSBBs were allowed), and improved and maintained liver function [defined as improvements in albumin, bilirubin, and International Normalized Ratio (INR) resulting in Child–Pugh A]. Removal of the underlying etiology was not included in the recompensation definition due to the challenge of completely eliminating metabolic dysfunction and the absence of a standardized definition of controlled etiology in patients with MASLD.

Secondary outcomes included an extended criteria for recompensation, considering recompensated patients those who achieved the aforementioned criteria for >12 months while maintaining a minimum and stable dose of treatment (ie, furosemide ≤25 mg/d, spironolactone ≤200 mg/d, and lactulose <2 packets/d).^13^ In addition to the primary events of interest, incidence of transjugular intrahepatic portosystemic shunt (TIPS) placement, HCC, mortality, and liver transplantation were recorded. Patients who underwent TIPS placement were censored from de analysis, as TIPS may independently influence clinical outcomes without being part of the predefined criteria for recompensation. Death was classified as liver-related if it was a consequence of liver failure or HCC. Liver failure-related death was considered if it occurred as a direct consequence of the progression of the underlying disease, whereas HCC-related death was defined as death resulting from complications directly attributable to HCC, including tumor progression or treatment failure. The cause of death was adjudicated based on the clinical judgment of the investigators and clinical reports. The impact of recompensation on all-cause and liver-related mortality was also evaluated as a secondary outcome.

### Ethics

This study was approved by the local IRB (PR (AG)621/2021)at all participating centers and was conducted in compliance with the Declaration of Helsinki and Istanbul. All patients provided written informed consent to participate. Confidentiality was preserved in agreement with current Spanish legislation on data protection (article 9 of the EU legislation 2016/679).

### Statistical analysis

Normally distributed variables were reported as means±SD and non-normally distributed variables as median and IQR. Normal distribution was assessed using the Kolmogorov–Smirnov method. Comparative analyses were performed using a two-tailed Student *t* test for continuous variables normally distributed or a Mann–Whitney test for continuous variables not normally distributed. Categorical variables were compared using the chi-square test or the Fisher's exact test, the latter being used in small sample situations.

Survival analyses were performed using competing risks models. The cumulative incidence function (CIF) was used to estimate the probability of experiencing liver recompensation over time, considering all-cause death and liver transplantation as competing events. The Fine and Gray subdistribution hazard model was applied for multivariate analyses to assess the impact of various covariates on the risk of recompensation. Extended recompensation criteria were used for the univariate and multivariate analyses, as the number of outcomes was higher and the characteristics of patients meeting the standard or extended recompensation criteria were comparable. Subhazard ratios (SHR) and their 95% CI for the occurrence of the primary endpoint in the presence of the competing risk were provided. All variables clinically and statistically significant (*p* value <0.1) were considered for multivariate analysis. The number of variables included in the initial models followed the rule of one variable per 10 outcomes. To avoid collinearity, we did not include variables in scores in the same model.

The impact of recompensation on overall mortality was analyzed according to a cause-specific Cox model, considering recompensation as a time-varying variable. Patients were censored at the time of LT or TIPS placement. Competing risk analysis was not feasible for this analysis, as incorporating time-dependent covariates can lead to distorted results.^14^ We introduced, therefore, a Cox cause-specific model for the analysis of predictors of LT, which is a competing event for mortality. Hazard ratios (HR) and their 95% confidence intervals (CI) were calculated. Age, Model for End-Stage Liver Disease—Sodium (MELD-Na), albumin, body mass index (BMI), total cholesterol and triglycerides, alcohol consumption, as well as recompensation were considered time-varying variables.

The impact of recompensation on each terminal event (eg, liver failure-related mortality, HCC-related mortality) was evaluated using the Fine and Gray subdistribution hazard model, with all other terminal events considered as competing risks.

All confidence intervals (CIs) and significance tests were two-sided with a level of significance of 0.05. All statistical analyses were conducted using STATA/BE and R version 4.1.0 (R Foundation for Statistical Computing, Vienna, Austria). The “cmprsk” package was utilized for the competing risks analysis, and the “survival” package was used for traditional survival analysis methods.

## RESULTS

### Characteristics of the study population

**FIGURE 1:**
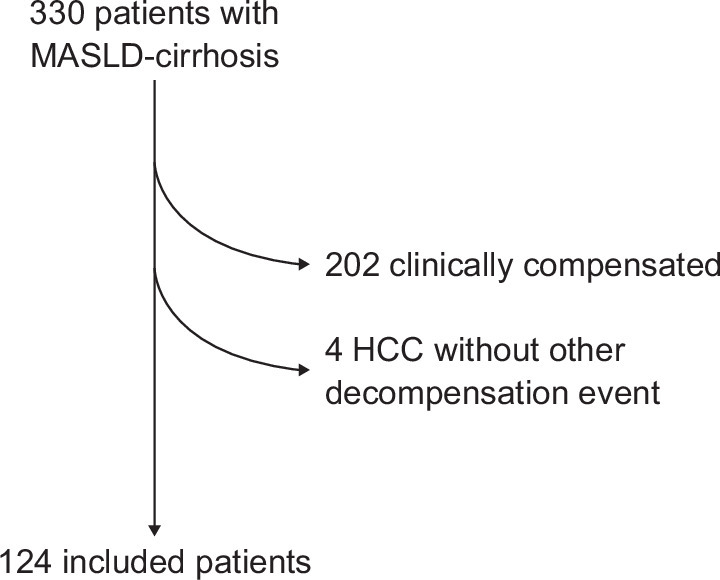
Patients’ flowchart. Abbreviations: HCC, hepatocellular carcinoma; MASLD, metabolic dysfunction–associated steatotic liver disease.

A total of 333 patients with MASLD cirrhosis were included in the registry. Of those, 124 patients decompensated during follow-up, met all inclusion criteria, did not present any exclusion criteria, and were subsequently included in the study (Figure 1). Baseline characteristics of patients at the time of their first decompensation are shown in Table 1. Sixty-six (53%) were male, with a median age of 69 years (IQR 62–73). Obesity was present in 59% of the patients, and T2D in 74%. Of the patients included in the study, less than a third presented active alcohol consumption, with an average consumption of 7 standard drinks per week (IQR 4–14). At the time of decompensation, the majority of patients were Child–Pugh class B (61%) with a median MELD-Na score of 11 (IQR 10–16).

**TABLE 1:** Baseline characteristics at decompensation of patients who achieved recompensation during follow-up compared with patients who did not

Patients	n=124 patients	Recompensation n=30	No recompensation n=94	*p*
Age (y)	69 (62–73)	68 (61–72)	69 (63–74)	0.296
Sex—male	66 (53%)	14 (48%)	52 (55%)	0.408
Current low-risk alcohol consumption	36 (29%)	9 (30%)	27 (29%)	0.582
Weekly standard drinks among consumers (n=36)	7 (4–14)	7 (3–11)	7 (5–14)	0.421
Current smoker	17 (14%)	6 (20%)	11 (12%)	0.338
BMI (kg/m^2^) (n=114)	32.2±5.6	32.5±6.1	32.2±5.5	0.834
Adjusted BMI (kg/m^2^) (n=114)^a^	32.2±5.6	32.5±6.1	32.0±5.5	0.834
Normal weight	18 (15%)	6 (20%)	12 (13%)	0.374
Overweight	33 (27%)	5 (17%)	28 (30%)	0.157
Obesity	73 (59%)	19 (63%)	54 (57%)	0.568
Arterial hypertension	82 (66%)	20 (67%)	62 (66%)	0.943
Type 2 diabetes mellitus	92 (74%)	25 (83%)	67 (71%)	0.189
Dyslipidemia	58 (47%)	13 (43%)	45 (48%)	0.664
Previous stroke	3 (2%)	1 (3%)	2 (2%)	0.708
Ischemic heart disease	18 (15%)	4 (13%)	14 (15%)	0.833
Leukocytes (10^9^/L)	4.7 (3.8–6.5)	5.1 (4.0–6.7)	4.6 (3.6–6.1)	0.188
Bilirubin (mg/dL)	1.2 (0.9–1.8)	1.15 (0.8–1.7)	1.3 (0.9–1.8)	0.170
Albumin (g/L)	3.30±0.63	3.53±0.67	3.23±0.61	0.023
INR	1.25 (1.12–1.44)	1.2 (1.2–1.3)	1.3 (1.1–1.5)	0.321
Platelets (10^9^/L)	91 (67.5–130.5)	93 (68–132)	91 (67–130)	0.743
Glycated hemoglobin (%) (n=49)	6 (5.3–6.7)	6 (5.6–6.5)	6 (5.3–6.8)	0.871
Total cholesterol (mg/dL)	137±40.2	128±28.2	141±43.0	0.183
LDL-cholesterol (mg/dL)	77±26.4	69±16.3	79±28.7	0.320
HDL-cholesterol (mg/dL)	43±15.8	45±15.1	42±16.2	0.624
Triglycerides (mg/dL)	96 (76–127)	99 (81–149)	95 (76–123)	0.283
Alanine aminotransferase (U/L)	30 (19–44)	31 (22–41)	30 (19–44)	0.848
Aspartate aminotransferase (U/L)	45 (36–62)	40 (36–54)	46 (37–65)	0.310
Alkaline phosphatase (U/L)	122 (87–183)	109 (78–148)	127 (94–188)	0.077
Gamma glutamyltransferase (U/L)	80 (52–192)	71 (54–124)	85 (47–210)	0.593
Creatinine (mg/dL)	0.84 (0.62–1.13)	0.82 (0.62–0.93)	0.85 (0.62–1.2)	0.220
Sodium (mmol/L)	139 (137–141)	139 (138–141)	140 (137–141)	0.730
Child–Pugh score (points)	7 (7–8)	7 (6–8)	8 (7–9)	0.001
A (5–6)	32 (26%)	14 (47%)	18 (19%)	
B (7–9)	76 (61%)	16 (53%)	50 (64%)	
C (10–15)	16 (13%)	0 (0%)	16 (17%)	
MELD-Na	11 (10–16)	11 (10–13)	12 (10–18)	0.063
FIB-4	6.88 (4.39–9.47)	5.25 (3.13–7.12)	7.15 (5.22–10.61)	0.006
Liver stiffness (kPa) (n=39)	26.8 (19.4–48)	25.7 (21.3–56.8)	27.0 (18.7–48.0)	0.939
CAP (dB/m) (n=26)	290±52.3	312±38.8	280±55.6	0.128
Endoscopic signs of portal hypertension (n=89)	76 (86%)	23 (92%)	53 (83%)	0.270
Gastroesophageal varices (n=89)	52 (58%)	17 (68%)	35 (55%)	0.252
Portal hypertensive gastropathy (n=89)	42 (47%)	13 (57%)	29 (55%)	0.570
High risk varices (n =89)	22 (25%)	5 (10%)	17 (27%)	0.519
Radiographic signs of portal hypertension (n=117)	97 (83%)	23 (79%)	74 (84%)	0.553
HVPG (mm Hg) (n=25)	17±4.0	18±2.1	17±4.3	0.703
Median disease time (y)	2.6 (0.5–5.0)	1.5 (0–4.5)	3 (1–5.5)	0.052
≥2 acute decompensations	26 (21%)	0 (0%)	26 (28%)	0.001
Type of first decompensation				
Ascites	89 (72%)	12 (40%)	77 (82%)	<0.001
Hepatic encephalopathy	29 (23%)	7 (23%)	22 (23%)	0.994
Portal hypertension-related bleeding	26 (21%)	11 (37%)	15 (16%)	0.015
Statins	50 (40%)	14 (47%)	36 (39%)	0.440
Pioglitazone	5 (4%)	2 (7%)	3 (3%)	0.595
GLP1r-a	4 (3%)	2 (7%)	2 (2%)	0.230
SGLT2i	6 (5%)	1 (3%)	5 (5%)	0.644
NSBBs	40 (33%)	8 (27%)	32 (34%)	0.431

^a^ Adjusted BMI was calculated using a dry weight estimation calculated by subtracting a percentage of body weight based on fluid retention severity (5% for mild ascites, 10% for moderate, 15% for severe, and an additional 5% for bilateral pedal edema to the knees)

Abbreviations: BMI, body mass index; CAP, controlled attenuation parameter; FIB-4, FIB-4, Fibrosis-4 index; GLP1r-a, glucagon-like peptide-1 receptor agonists; HDL, high-density lipoprotein; HVPG, hepatic venous-portal gradient; INR, International Normalized Ratio; LDL, low-density lipoprotein​​​​​; MELD-Na, Model for End-stage Liver Disease—Sodium; NSSBs: Non-selective β-blockers; SGLT2i: Sodium-glucose transport protein 2 inhibitors.

Among those patients who had undergone recent gastroscopy, almost all (86%) exhibited endoscopic signs of portal hypertension, with 52 patients having gastroesophageal varices. Imaging signs of portal hypertension were identified in 83% of the patients. HVPG measurement was performed in 25 patients (20%), with a median HVPG value of 17 mm Hg (SD±4.0). The median LSM was 26.8 kPa (IQR 19.4–48), with a CAP of 290 dB/m (SD 52.3).

### Characteristics of the first decompensation event

Ascites was the most frequent hepatic decompensation in 89 out of 124 (72%), followed by hepatic encephalopathy (29 patients, 23%), and PHB (26 patients, 21%). Less than a quarter of patients (21%) experienced more than one type of liver decompensation as the first decompensating event (Table 1).

### Hepatic recompensation

During the study period, 30 patients (24%) and 51 patients (42%) fulfilled the recompensation and extended recompensation criteria, respectively. At baseline (Table 1), patients who recompensated during follow-up showed better baseline liver function, as assessed by Child–Pugh score [7 points (IQR 6–8) vs. 8 (IQR 7–9); *p*=0.001] with a trend toward lower values of MELD-Na score [11 (IQR 10–13) vs. 12 (IQR 10–18); *p*=0.063], a more recent disease diagnosis [1.5 years (IQR 0–4.5) vs. 3 (IQR 1–5.5); *p*=0.052], higher albumin (3.53±0.67 vs. 3.23±0.61; *p*=0.023) and lower median FIB-4 score [5.25 (IQR 3.13–7.12) vs. 7.15 (IQR 5.22–10.61); *p*=0.006].

Ascites was more commonly observed in patients who did not achieve recompensation during follow-up (82% vs. 40%; *p*<0.001), while PHB was the predominant form of decompensation in patients who did recompensate (37% vs. 16%; *p*=0.015). Similar data were observed in patients who met the extended recompensation criteria (information available in Supplemental Table S1, http://links.lww.com/HC9/C264).

Low-risk alcohol consumption was not significantly related to the occurrence of hepatic recompensation either at the time of decompensation or during the follow-up period.

Similarly, the use of relevant co-medication [statins, glucagon-like peptide-1 receptor agonists (GLP1r-a), and sodium/glucose cotransporter 2 inhibitors (SGLT2i)] was not significantly associated with hepatic recompensation (Table 1).

### Determinants of recompensation

**FIGURE 2:**
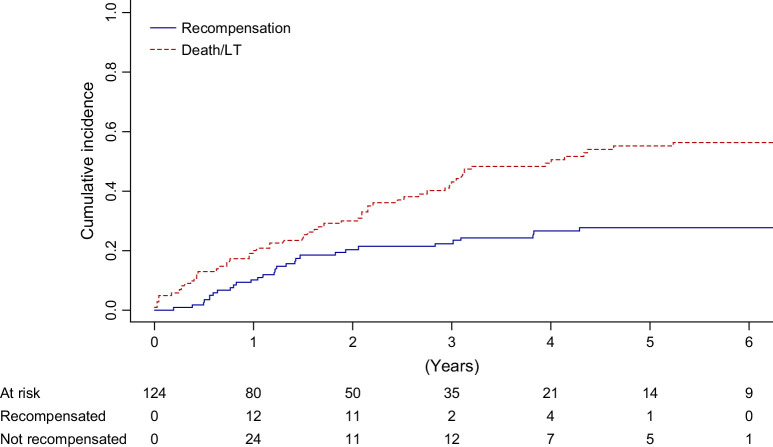
Cumulative incidence curves for recompensation and death/liver transplantation. Abbreviation: LT, liver transplantation.

Considering all-cause death and liver transplantation as competing events, the cumulative incidence of recompensation was 13% at 1 year, 24% at 2 years, and 31% at 4 years (Figure 2). The cumulative incidence of death or liver transplantation was 26% at 1 year, 37% at 2 years, and 57% at 4 years. The cumulative incidence of recompensation with extended criteria was 23% at 1 year, 43% at 2 years, and 47% at 4 years (Supplemental Figure S1, http://links.lww.com/HC9/C264).

In the univariable competing risk regression analysis for standard (Supplemental Table S2, http://links.lww.com/HC9/C264) and extended recompensation (Table 2), better liver function tests, lower MELD-Na and Child–Pugh scores, were associated with a higher probability of recompensation, whereas ascites, PHB, or multiple decompensations as the first decompensating event were associated with a lower probability of recompensation. Multivariable analysis of predictors of recompensation identified lower MELD-Na, albumin, and type of first decompensation (ascites and PHB or multiple decompensations) as independent predictors of recompensation.

**TABLE 2:** Predictors of extended hepatic recompensation

	Univariable analysis				Multivariable analysis		
	SHR	95% CI	*p*		aSHR	95% CI	*p*
				**Model 1**			
Age (y)	0.980	0.956–1.006	0.123	Age (y)	0.997	0.961–1.035	0.884
Sex—female	1.581	0.913–2.737	0.102	MELD-Na	0.891	0.833–0.953	0.001
Low-risk alcohol consumption	0.742	0.409–1.345	0.325	Albumin (g/L)	1.894	1.088–3.297	0.024
Adjusted BMI^a^ (kg/m^2^)	0.998	0.944–1.054	0.930	Platelets (10^9^/L)	1.00	0.999–1.006	0.118
Glycated hemoglobin (%)	1.028	0.845–1.251	0.779	Ascites	0.475	0.268–0.841	0.011
Total cholesterol (mg/dL)	1.006	0.999–1.012	0.074				
Triglycerides (mg/dL)	1.001	0.997–1.006	0.530				
Platelets (10^9^/L)	1.003	0.999–1.006	0.092	**Model 2**			
INR	0.212	0.071–0.630	0.005	Age (y)	0.972	0.942–1.003	0.073
Albumin (g/L)	2.111	1.308–3.407	0.002	MELD-Na	0.903	0.836–0.975	0.009
Bilirubin (mg/dL)	0.715	0.562–0.909	0.006	Albumin (g/L)	1.74	1.040–2.926	0.035
Creatinine (mg/dL)	0.325	0.173–0.614	0.001	Platelets (10^9^/L)	1.002	0.999–1.004	0.290
Child–Pugh score (points)	0.703	0.587–0.843	<0.001	Multiple decompensation	0.151	0.033–0.698	0.015
MELD-Na	0.860	0.801–0.924	<0.001				
Multiple decompensation	0.123	0.029–0.528	0.005				
Ascites	0.547	0.316–0.948	0.032				
Hepatic encephalopathy	0.711	0.335–1.510	0.375				
Portal hypertension-related bleeding	1.263	0.702–2.270	0.437				
Statins	0.789	0.446–1.395	0.415				
Pioglitazone	2.606	0.723–9.396	0.143				
GLP1r-a	1.171	0.272–5.050	0.832				
SGLT2i	0.903	0.176–4.630	0.902				
NSBBs	0.936	0.506–1.731	0.833				

^a^ Adjusted BMI was calculated using a dry weight estimation calculated by subtracting a percentage of body weight based on fluid retention severity (5% for mild ascites, 10% for moderate, 15% for severe, and an additional 5% for bilateral pedal edema to the knees).

Abbreviations: aSHR, adjusted subhazard ratio; BMI, body mass index; FIB-4, Fibrosis-4 index; GLP1r-a, glucagon-like peptide-1 receptor agonists; INR, International Normalized Ratio; MELD-Na, Model for End-stage Liver Disease—Sodium; NSSBs, non-selective β-blockers; SGLT2i, sodium-glucose transport protein 2 inhibitors; SHR, subhazard ratio.

In a subanalysis excluding patients with preserved liver function at the time of first decompensation (defined by Child–Pugh A), factors associated with recompensation were largely comparable to those identified in the overall cohort (Supplemental Table S3, http://links.lww.com/HC9/C264), with 2 notable exceptions: (1) type of first decompensation event was no longer associated with the probability of recompensation and (2) platelet count was positively associated with the likelihood of recompensation.

### Metabolic risk factors

**FIGURE 3:**
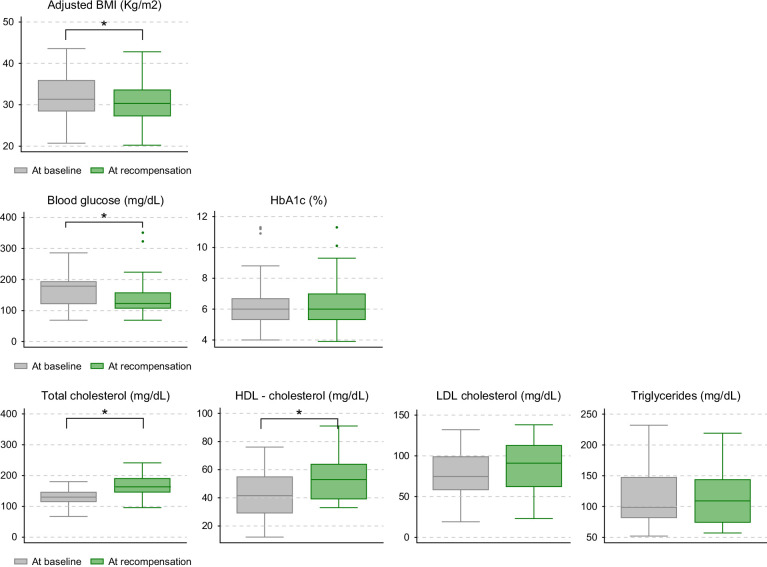
Changes in metabolic variables from the time of decompensation to the time of recompensation.

Paired Student’s *t* tests were performed to evaluate changes in metabolic variables from the time of decompensation to the time of recompensation. BMI decreased significantly from baseline to recompensation (33.4±6.1 vs. 30.8±5.6 kg/m^2^; mean reduction 2.6 kg/m^2^, *p*=0.010). A non-significant trend toward improved glycemic control was observed (fasting glucose 185±62.5 vs. 125±60.6 mg/dL; *p*=0.072), with stable HbA1c levels. An increase in total cholesterol (129±28.5 vs. 175±46.2 mg/dL; *p*<0.01) and HDL-cholesterol (41.1±30.5 vs. 58.1±20.8 mg/dL; *p*=0.040) was observed at recompensation, while triglycerides and LDL-cholesterol showed no significant changes (Figure 3).

### Survival analysis

The median follow-up time from the first decompensation event to the terminal event (death, liver transplantation, or last recorded follow-up) was 2.1 years (IQR 0.97–4.53). During that time, we observed 59 deaths (48%), and 18 (15%) patients received a liver transplant. The detailed causes of mortality in our cohort are shown in Table 3. The majority of deaths were liver-related (80%), that is, 36 (61%) due to liver failure or direct complications of cirrhosis, and 11 (19%) related to HCC. None of the patients included in the study died from a cardiovascular disease.

**TABLE 3:** Deaths and liver transplantation frequencies of the patients included according to recompensation

Patients	n=124 patients	Recompensation n=30	No recompensation n=94	*p*
Deaths	59 (48%)	8 (27%)	51 (54%)	0.008
Liver-related deaths	47 (80%)	4 (13%)	43 (46%)	
Liver failure	36 (61%)	2 (25%)	34 (67%)	
Hepatocellular carcinoma	11 (19%)	2 (25%)	9 (18%)	
Extrahepatic cancer	1 (1%)	0 (0%)	1 (2%)	
Cardiovascular disease	0 (0%)	—	—	
Others	11 (19%)	4 (50%)	7 (14%)	
Liver transplant (LT)	18 (15%)	5 (17%)	13 (14%)	0.767
Liver failure	12 (67%)	3 (60%)	9 (69%)	
Hepatocellular carcinoma	6 (33%)	2 (40%)	4 (31%)	

**TABLE 4:** Predictors of all-cause mortality using the Cox proportional hazard model with time-dependent covariates

	Univariable analysis		Multivariable analysis		
Patients	HR (95%CI)	*p*		aHR (95% CI)	*p*
Age (y)	1.082 (1.048–1.117)	<0.001	**Model 1**		
Sex—male	0.816 (0.488–1.363)	0.437	Recompensation	0.248 (0.028–2.226)	0.213
Low-risk alcohol consumption	0.879 (0.339–2.280)	0.791	MELD-Na	1.211 (1.165–1.258)	<0.001
Adjusted BMI* (kg/m^2^) (n=106)	0.842 (0.765–0.926)	<0.001			
Glycated hemoglobin (%)	0.739 (0.318–1.715)	0.481	**Model 2**		
Total cholesterol (mg/dL)	0.967 (0.958–0.976)	<0.001	Age (y)	1.100 (1.001–1.200)	0.034
Triglycerides (mg/dL)	0.981 (0.970–0.993)	0.002	Adjusted BMI (kg/m^2^) (n=106)	0.937 (0.815–1.080)	0.367
Bilirubin (mg/dL)	1.224 (1.145–1.310)	<0.001	Total cholesterol (mg/dL)	1.001 (0.983–1.019)	0.889
Albumin (g/L)	0.141 (0.085–0.234)	<0.001	Albumin (g/L)	0.432 (0.109–1.710)	0.232
INR	2.25 (1.552–3.267)	<0.001	MELD-Na	1.404 (1.181–1.669)	<0.001
Platelets	0.996 (0.991–1.001)	0.169			
Creatinine (mg/dL)	1.496 (1.321–1.696)	<0.001			
Child–Pugh score (points)	2.453 (2.020–2.980)	<0.001	**Model 3**		
MELD-Na	1.218 (1.172–1.264)	<0.001	Age (y)	1.079 (1.030–1.131)	0.001
Recompensation	0.080 (0.011–0.581)	0.013	Adjusted BMI (kg/m^2^) (n=106)	0.940 (0.790–1.004)	0.279
Extended recompensation	0.042 (0.006–0.304)	0.002	Total cholesterol (mg/dL)	0.972 (0.961–0.983)	<0.001
			Albumin (g/L)	0.381 (0.184–0.810)	0.012
			Recompensation	0.425 (0.051–3.520)	0.427

* Adjusted BMI was calculated using a dry weight estimation calculated by subtracting a percentage of body weight based on fluid retention severity (5% for mild ascites, 10% for moderate, 15% for severe, and an additional 5% for bilateral pedal edema to the knees).

Abbreviations: aHR, adjusted hazard ratio; BMI, body mass index; HR, hazard ratio; INR, International Normalized Ratio; MELD-Na, Model for End-stage Liver Disease—Sodium.

**FIGURE 4:**
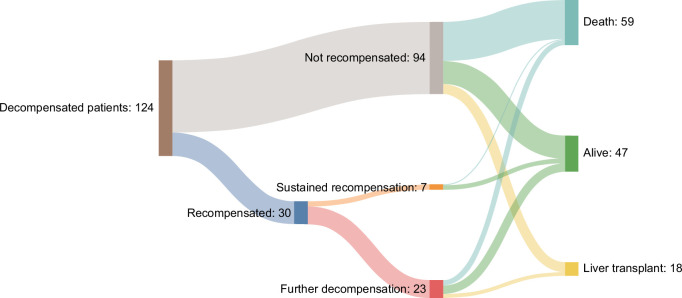
Sankey plot reporting major clinical outcomes of the patients included in the study according to recompensation and further decompensation (redecompensation).

Among patients who achieved recompensation according to standard criteria, only 7 (23%) remained stably recompensated throughout follow-up, while the rest experienced subsequent decompensation after a median of 1.7 years (IQR 0.8–4.3). Median time during which patients remained in a recompensated status was 1.48 years (IQR 1.0–4.0) for standard criteria and 1.43 years (IQR 0.96–4.12) for extended criteria. At the end of follow-up, all but 1 patient with sustained recompensation were alive, and none of them needed a liver transplant. Of note, the single death in this group was not liver-related. In contrast, most deaths and liver transplantations occurred in patients who either never achieved recompensation or developed recurrent decompensation after an initial recompensated phase (Figure 4).

Liver-related mortality was lower in patients who recompensated according to standard criteria, regardless of liver function when stratified by MELD-Na or Child–Pugh categories (Supplemental Table S5, S6, http://links.lww.com/HC9/C264). The positive effect on survival was primarily driven by a reduction in liver-related mortality due to liver failure or disease progression (SHR: 0.144, 95% CI [0.364–0.570]; *p*=0.006) (Supplemental Figure S2B, http://links.lww.com/HC9/C264). Recompensation did not affect liver transplantation, HCC-related mortality, or mortality due to other causes.

In univariable Cox proportional hazard analysis, recompensation was associated with a significantly lower risk of all-cause mortality (HR 0.080, 95% CI [0.011–0.581]; *p*=0.013). In addition, impaired liver and renal function—reflected by higher bilirubin, INR, creatinine, Child–Pugh score, and MELD-Na—were strongly associated with mortality risk. Conversely, higher values of BMI, total cholesterol, triglycerides, and albumin—parameters commonly interpreted as indicators of preserved nutritional and synthetic status in advanced liver disease—were associated with a lower risk of mortality. However, in the multivariable analysis, recompensation was no longer independently associated with survival after adjustment for age, BMI, total cholesterol, albumin, and particularly MELD-Na. When considering recompensation as a time-dependent covariate, liver function emerged as the most important determinant of all-cause mortality, with MELD-Na showing the strongest prognostic factor (aHR 1.404, 95% CI 1.181–1.669; *p*<0.001). These findings were consistent when restricting the analysis to liver-related mortality or when applying extended criteria for recompensation (Table 4).

## DISCUSSION

In this study, we describe for the first time the natural history of patients with MASLD-related decompensated cirrhosis in a real-life setting, focusing on the phenomenon of recompensation. From our results, we can draw 4 main preliminary conclusions that warrant further validation: (1) Recompensation in MASLD-cirrhosis is possible with the highest probability of achieving recompensation in the period shortly after the first decompensation event. (2) “Extended” recompensation criteria seem to largely overlap with those proposed by the Baveno VII consensus. (3) Liver function appears to be a major determinant of both the likelihood of recompensation and overall mortality. (4) The majority of recompensated patients experience further decompensation during follow-up.

Since the 2022 Baveno VII consensus criteria first included the concept of recompensation,^11^ there has been significant debate within the scientific community. This definition embodies previous findings suggesting that certain patients with decompensated cirrhosis can clinically improve to a state free from overt signs of decompensation, coupled with enhanced liver function parameters. This reversal of disease progression, primarily observed in conjunction with the elimination of the underlying etiology, represents a ground-breaking and relatively unexplored area in the management of MASLD cirrhosis. In our study, hepatic recompensation occurred with a cumulative incidence of 13% at 1 year and 24% at 2 years. The proportion of patients fulfilling recompensation criteria is comparable to data from studies focusing on other etiologies of cirrhosis, such HCV, HBV, and ALD patients. The literature on the topic is still scarce, primarily focusing on well-defined and easily removable or controlled etiologies, namely ALD and viral hepatitis. Studies from the delisting of liver transplant candidates suggest that sustained alcohol abstinence in ALD cirrhosis improves prognosis, with a subset of patients experiencing recompensation (8.6%–16.5%).^15^,^16^ Direct-acting antiviral therapies have significantly altered the course of HCV cirrhosis, with many patients regressing to a compensated stage (31.6% of Child–Pugh class B and 12.3% of Child–Pugh class C patients over a median observation period of 11 months.^17^ Similarly, long-term antiviral treatment with entecavir in HBV decompensated cirrhosis has demonstrated substantial rates of recompensation (56.2%), validating the Baveno criteria in treatment-naïve patients.^18^ In a recent systematic review and meta-analysis by Goh et al,^19^ the pooled prevalence of recompensation across etiologies was ~35%, with lower rates observed in non-curable etiologies, such as autoimmune hepatitis or primary biliary cholangitis (PBC). Specifically, patients with autoimmune hepatitis–related cirrhosis exhibited a recompensation prevalence of ~25% (95% CI 4%–30%), which is remarkably similar to the 24% observed in our study.

In order to evaluate recompensation in MASLD patients, we had to adapt the definition proposed by Baveno VII and remove the condition related to the underlying etiology. To date, there is no consensus regarding etiological control nor approved treatments to prevent disease progression in MASLD cirrhosis. We were particularly interested in whether patients with decompensated MASLD cirrhosis can achieve hepatic recompensation in the absence of a strict etiological control. By applying objective criteria—improvement or normalization of bilirubin, albumin, and INR, alongside resolution of decompensating events without prescribed specific treatment, we identified almost a quarter of patients who fulfilled recompensated criteria at some point after the first decompensation.

When assessing predictors of recompensation, baseline liver function and the nature of the decompensation event emerged as the strongest determinants. In contrast, we found a lack of correlation between baseline metabolic risk factors and recompensation. Interestingly, despite the lack of association at baseline, improvement in some well-established metabolic risk factors was observed in patients who achieved recompensation. Specifically, reductions in body weight and BMI, together with increases in HDL-cholesterol, were noted when compared with baseline values. Conversely, other metabolic markers, such as total cholesterol, LDL-cholesterol, and triglycerides, remained unchanged or even increased over time. These seemingly discordant findings prompt the question of how cardiometabolic risk factors perform once an advanced decompensated stage of liver disease is reached, and if this is comparable to earlier stages of the disease. Whether hepatic recompensation can be actively promoted through metabolic optimization, therefore, remains uncertain. Two major challenges may account for this uncertainty. First, there is a lack of standardized, disease-stage-specific cutoff values to define adequate metabolic control in patients with decompensated MASLD-related cirrhosis. Second, several parameters commonly used to assess metabolic risk are intrinsically altered in cirrhosis, regardless of etiology, including lipid levels and arterial blood pressure, which are strongly influenced by impaired hepatic synthetic function and systemic circulatory changes. In this context, higher cholesterol levels may paradoxically reflect improved hepatic synthetic capacity or nutritional status rather than worsening cardiometabolic risk.

Taken together, these findings underscore the need to establish cirrhosis stage–specific metabolic thresholds and to determine whether sustained improvements in glycemic control, body composition, and lipid metabolism can meaningfully increase the likelihood or durability of recompensation. Consistently, none of the metabolic therapies evaluated in our study showed a significant association with recompensation probability, further highlighting the complexity of disentangling the role of metabolic control from advanced liver disease severity.

Less than a third of patients had active alcohol consumption (in any case exceeding the threshold definition of MASLD)^1^ at the time of their first decompensation, although average consumption was low. Active alcohol consumption at the time of decompensation was not significantly associated with the likelihood of subsequent recompensation. It is worth noting that all patients who achieved recompensation ceased alcohol consumption during follow-up, and persistent alcohol consumption was only observed in patients who did not achieve recompensation. However, alcohol consumption was documented based on medical records and self-reported by patients, with no objective measurement of consumption. Because self-reported alcohol intake is known to be unreliable, some patients classified as MASLD may actually fulfill criteria for MetALD. Therefore, we cannot exclude the possibility that complete and sustained alcohol cessation, even in patients with lower consumption, may influence the likelihood of recompensation.^20^

In our cohort, neither the classical indirect nor direct measurements of portal hypertension were found to be associated with recompensation. However, the limited number of patients who underwent LSM and/or HVPG restricts the generalizability of these findings.

Interestingly, the preliminary conclusion that the clinical presentation of first decompensation appears to be a critical determinant in the likelihood of recompensation can be drawn. In particular, patients presenting with ascites—especially when accompanied by additional decompensating events—face substantially lower odds of meeting both simple and extended recompensation criteria. It is reasonable to assume that patients presenting with variceal bleeding are more likely to achieve recompensation, since they usually exhibit better preserved liver function parameters and their clinical management does not require the withdrawal of other disease-specific therapies, such as diuretics in patients with ascites or lactulose/rifaximin in patients with hepatic encephalopathy. The discontinuation of these treatments is left to clinical judgment and is not guided by standardized recommendations, which may often result in delayed or incomplete withdrawal. Are patients decompensating from hypertension-related bleeding comparable to those presenting with hepatic encephalopathy or ascites in terms of the likelihood of achieving recompensation? This raises a broader issue beyond the scope of our study, suggesting the need for a reconsideration and potential revision of recompensation criteria, regardless of the underlying liver disease etiology.

Ours is not the first study to propose expanded criteria for recompensation. Tonon et al.^13^ recently proposed to include in the definition those patients with either ALD or viral cirrhosis who achieve significant liver function and/or clinical improvement with low treatment doses. In our study, baseline characteristics at the time of the first decompensation, as well as disease progression and prognosis, were comparable between patients who met the adapted Baveno criteria and those who met the extended criteria. Thus, the positive effect of recompensation may also occur in patients receiving low-dose treatment, raising questions about whether this criterion should be strictly applied when determining whether a patient is considered recompensated.

Our study did not demonstrate a definitive survival benefit from recompensation. Although patients who achieved recompensation initially appeared to be at lower risk of liver-related death or transplantation, this effect was not independent once recompensation was modeled as a time-dependent covariate, particularly when adjusted for MELD-Na. Only 23% of recompensated patients remained clinically recompensated during follow-up, underscoring that the protective effect of recompensation is lost once further decompensation occurs and that liver function remains the dominant predictor of survival in these cases. In contrast, when recompensation was treated as a non-time-dependent covariate, a favorable association emerged (Supplemental Table S7, http://links.lww.com/HC9/C264). Importantly, none of the patients with sustained recompensation required transplantation or died from liver-related causes, suggesting that recompensation is possible and likely beneficial.

This apparent discrepancy may be explained by the progressive course of MASLD-related cirrhosis, for which no disease-modifying therapy is currently available. Thus, for recompensation to exert a meaningful impact on long-term outcomes, it likely needs to occur in parallel with effective disease control or suppression of the underlying etiology.

Our study has some limitations that need to be acknowledged. First, the partial retrospective design presents inherent challenges, particularly with the reliance on electronic health records, which limited the availability of certain variables. Second, evolving diagnostic criteria for MASLD cirrhosis during this timeframe could have introduced variability. In addition, the use of self-reported alcohol consumption may have influenced the accurate classification of patients across the SLD spectrum, as noted earlier. However, the extended follow-up of patients helped reinforce diagnostic accuracy and excluded other suspected etiologies with greater certainty. On the other hand, this prolonged recruitment period resulted in an underrepresentation of more recently introduced pharmacological therapies of potential interest, such as aGLP1 and iSGLT2, as well as NSBBs, as primary prophylaxis for liver decompensation. This underrepresentation precluded a comprehensive analysis of their potential effects on patients’ outcomes. Third, the relatively small cohort size represents another limitation, as it reduces statistical power, potentially hindering the detection of statistically significant associations despite observable trends. Yet, this is, to our knowledge, the largest cohort of patients with decompensated MASLD cirrhosis communicated thus far. Fourth, an additional limitation lies in the lack of strict control over disease drivers and the necessary adaptation of the Baveno VII criteria for recompensation. Specifically, the exclusion of etiological control or removal as a requisite for recompensation deviates from conventional standards. This adaptation reflects the current limited understanding of the natural history of MASLD cirrhosis and the absence of targeted therapies to halt disease progression, making it challenging to fully assess recompensation using established criteria.

In conclusion, patients with decompensated MASLD cirrhosis can recompensate, and this seems to occur at a non-negligible rate, and it seems to be strongly associated with liver function. In addition, patients who achieve recompensation show improvements in selected metabolic parameters. However, most patients who achieve recompensation eventually experience further decompensation, which may be attributable to insufficiently sustained control of the underlying etiology. Future prospective studies with larger patient cohorts and extended follow-up periods are necessary to validate these findings and further elucidate the mechanisms and predictors of recompensation in patients with MASLD cirrhosis.

## Supplementary Material
