# Supplementary material for: The rate and predictors of recompensation in patients with decompensated cirrhosis due to metabolic dysfunction–associated liver disease (MASLD)
Source: Hepatol Commun. 2026 Mar 11;10(4):e00919. doi: 10.1097/HC9.0000000000000919 (PMC12978826; doi:10.1097/HC9.0000000000000919)
Supplement: Supplementary file 1 [file hc9-10-e00919-s001.docx]

**Supplementary material**

**Table of contents**

**Table S1 Baseline characteristics at decompensation of patients who achieved extended recompensation during follow-up compared to patients who did _______________________________ 2**

**Table S2. Predictors of hepatic recompensation using standard criteria __________________________4**

**Table S3. Predictors of extended hepatic recompensation (only considering Child Pugh B or C patients)_______________________________________________________________________________5**

**Table S4. Deaths and liver transplantation frequencies of the patients included according to recompensation ________________________________________________________________________6**

**Table S5. Deaths and liver transplantation frequencies according to recompensation stratified by Child Pugh score categories __________________________________________________________________7**

**Table S6. Deaths and liver transplantation frequencies according to recompensation stratified by MELD-Na score categories _____________________________________________________________________ 8**

**Table S7. Predictors of transplant-free survival considering recompensation as a non-time- dependent covariate ______________________________________________________________________________9**

**Table S8. Predictors of liver transplantation considering recompensation as a time-dependent covariate _____________________________________________________________________________________10**

**Figure S1. Cumulative incidence curves for extended recompensation and death/liver transplantation _____________________________________________________________________________________11**

**Figure S2 Survival curves considering recompensation as non-time-dependent covariate__________12**

**Table S1. Baseline characteristics at decompensation of patients who achieved extended recompensation during follow-up compared to patients who did not.**

| Patients | n = 124 patients | Extended recompensation  n = 51 | No recompensation  n = 73 | p value |
| --- | --- | --- | --- | --- |
| Age (years) | 69 (62 -73) | 67 (61 – 73) | 70 (64 – 75) | 0.073 |
| Sex. Male | 66 (53%) | 22 (42%) | 44 (61%) | 0.038 |
| Current low-risk alcohol consumption | 36 (29%) | 12 (23%) | 24 (33%) | 0.421 |
| Weekly standard drinks among consumers (n = 36) | 7 (4-14) | 7 (3 – 12) | 7 (5 – 14) | 0.361 |
| Current smoker | 17 (14%) | 6 (12%) | 11 (15%) | 0.259 |
| BMI (Kg/m^2^) (n=114) | 32.2 ± 5.6 | 32.4 ± 5.9 | 32.3 ± 5.5 | 0.915 |
| Adjusted BMI (Kg/m^2^) (n=114) * | 32.2 ± 5.6 | 32.3 ± 5.9 | 32.3 ± 5.5 | 0.915 |
| - Normal weight | 18 (15%) | 10 (19%) | 8 (11%) | 0.205 |
| - Overweight | 33 (27%) | 10 (19%) | 23 (32%) | 0.114 |
| - Obesity | 73 (59%) | 32 (62%) | 41 (57%) | 0.608 |
| Arterial hypertension | 82 (66%) | 32 (62%) | 50 (69%) | 0.359 |
| Type 2 diabetes mellitus | 92 (74%) | 39 (75%) | 53 (74%) | 0.862 |
| Dyslipidemia | 58 (47%) | 19 (37%) | 39 (54%) | 0.052 |
| Previous stroke | 3 (2%) | 1 (2%) | 2 (3%) | 0.760 |
| Ischemic heart disease | 18 (15%) | 6 (12%) | 12 (17%) | 0.424 |
| Leucocytes (10^9^/L) | 4.7 (3.8 – 6.5) | 4.9 (4.1 – 6.7) | 4.6 (3.3 – 6.2) | 0.147 |
| Bilirubin (mg/dL) | 1.2 (0.9 – 1.8) | 1.2 (0.8 – 1.7) | 1.3 (0.9 – 2.0 | 0.148 |
| Albumin (g/L) | 3.30 ± 0.63 | 3.5 ± 0.64 | 3.2 ± 0.59 | 0.002 |
| INR | 1.25 (1.12 - 1.44) | 1.22 (1.1 – 1.3) | 1.26 (1.13 – 1.5) | 0.069 |
| Platelets (10^9^/L) | 91 (67.5 – 130.5) | 93 (68 – 133) | 90 (65 – 129) | 0.510 |
| Glycated hemoglobin (%) (n=49) | 6 (5.3 – 6.7) | 6 (5.6 – 7.0) | 5.7 (5.3 – 6.5) | 0.520 |
| Total cholesterol (mg/dL) | 137 ± 40.2 | 146 ± 40.2 | 131 ± 39.3 | 0.058 |
| LDL- cholesterol (mg/dL) | 77 ± 26.4 | 82 ± 25.2 | 69 ± 26.8 | 0.127 |
| HDL- cholesterol (mg/dL) | 43 ± 15.8 | 45 ± 15.6 | 40 ± 16.0 | 0.357 |
| Triglycerides (mg/dL) | 96 (76 – 127) | 118 (82 – 142) | 90 (71 – 112) | 0.020 |
| Alanine aminotransferase (U/L) | 30 (19 – 44) | 32 (22- 48) | 28 (18 – 42) | 0.149 |
| Aspartate aminotransferase (U/L) | 45 (36 – 62) | 43 (36 – 64) | 45 (37 – 61) | 0.922 |
| Alkaline phosphatase (U/L) | 122 (87 – 183) | 115 (81 – 179) | 127 (95 – 193) | 0.189 |
| Gamma glutamyltransferase (U/L) | 80 (52 – 192) | 72 (55 – 129) | 87 (46 – 213) | 0.556 |
| Creatinine (mg/dL) | 0.84 (0.62 – 1.13) | 0.76 (0.58 – 0.92) | 0.88 (0.63 – 1.35) | 0.010 |
| Sodium (mmol/L) | 139 (137 – 141) | 140 (138 – 142) | 139 (137 – 141) | 0.197 |
| Child – Pugh score (points) | 7 (7 – 8) | 7 (6 – 8) | 8 (7 – 9) | <0.001 |
| - A (5-6) | 32 (26%) | 18 (35%) | 14 (19%) |  |
| - B (7-9) | 76 (61%) | 31 (61%) | 45 (62%) |  |
| - C (10-15) | 16 (13%) | 3 (6%) | 13 (18%) |  |
| MELD-Na | 11 (10 – 16) | 11 (9 – 13) | 13 (10 – 20) | 0.001 |
| FIB-4 | 6.88 (4.39 – 9.47) | 5.8 (3.4 – 8.5) | 7.3 (5.5 – 10.7) | 0.021 |
| Liver stiffness (kPa) (n = 39) | 26.8 (19.4 – 48) | 25.7 (18.2 – 47.0) | 27.2 (20.6 – 48.0) | 0.621 |
| CAP (dB/m) (n=26) | 290 ± 52.3 | 305 ± 41.9 | 281 ± 57.5 | 0.242 |
| Endoscopic signs of portal hypertension (n = 89) | 76 (86%) | 39 (91%) | 37 (84%) | 0.354 |
| - Gastroesophageal varices (n=89) | 52 (58%) | 26 (59%) | 26 (58%) | 0.900 |
| - Portal hypertensive gastropathy (n=89) | 42 (47%) | 21 (48%) | 21 (48%) | 0.920 |
| - High risk varices (n =89) | 22 (25%) | 10 (23%) | 12 (27%) | 0.667 |
| Radiographic signs of portal hypertension (n=117) | 97 (83%) | 39 (77%) | 58 (88%) | 0.104 |
| HVPG (mmHg) (n=25) | 17 ± 4.0 | 16.4 ± 3.6 | 17.1 ± 4.3 | 0.670 |
| Median disease time (years) | 2.6 (0.5 – 5.0) | 2 (0 – 4.5) | 3.5 (1 – 6) | 0.020 |
| ≥2 acute decompensations | 26 (21%) | 3 (6%) | 23 (32%) | <0.001 |
| Type of first decompensation |  |  |  |  |
| - Ascites | 89 (72%) | 32 (62%) | 57 (79%) | 0.031 |
| - Hepatic encephalopathy | 29 (23%) | 10 (19%) | 19 (26%) | 0.353 |
| - Portal hypertension-related bleeding | 26 (21%) | 13 (25%) | 13 (18%) | 0.349 |
| Statins | 50 (41%) | 18 (35%) | 32 (44%) | 0.309 |
| Pioglitazone | 5 (4%) | 3 (6%) | 2 (3%) | 0.390 |
| GLP1r-a | 4 (3%) | 2 (4%) | 2 (3%) | 0.735 |
| SGLT2i | 6 (5%) | 2 (4%) | 4 (6%) | 0.666 |
| NSBBs | 40 (33%) | 15 (29%) | 25 (35%) | 0.536 |

BMI: Body Mass Index; INR: International Normalized Ratio; MELD: Model for end-stage liver disease; CAP; Controlled Attenuation Parameter; HVPG: Hepatic venous-portal gradient;

GLP1r-a: Glucagon-like peptide-1 receptor agonists; NSSBs: Non-selective β-blockers; SGLT2i: Sodium-glucose transport protein 2 inhibitors

*Adjusted BMI was calculated using a dry weight estimation calculated by subtracting a percentage of body weight based on fluid retention severity (5% for mild ascites, 10% for moderate, 15% for severe, and an additional 5% for bilateral pedal edema to the knees)

**Table S2. Predictors of hepatic recompensation using standard criteria**

|  | Univariable analysis | | |  | Multivariable analysis | | |
| --- | --- | --- | --- | --- | --- | --- | --- |
|  | **SHR** | **95% CI** | **p-value** |  | **aSHR** | **95% CI** | **p-value** |
|  |  |  |  | **MODEL 1** | | | |
| Age (years) | 0.984 | 0.952 – 1.016 | 0.333 | **MELD-Na** | 0.877 | 0.783 – 0.981 | 0.021 |
| Sex. female | 1.200 | 0.588 - 2.448 | 0.617 | **Albumin (g/L)** | 1.74 | 1.040 – 2.926 | 0.035 |
| Low-risk alcohol consumption | 1.152 | 0.528 – 2.512 | 0.721 | **Total cholesterol (mg/dL)** | 0.987 | 0.978 – 0.997 | 0.008 |
| Adjusted BMI (Kg/m^2^) | 1.002 | 0.927 – 1.083 | 0.950 | **MODEL 2** | | | |
| Glycated hemoglobin (%) | 1.022 | 0.771 – 1.356 | 0.876 | **MELD-Na** | 0.950 | 0.874 – 0.985 | 0.028 |
| Total cholesterol (mg/dL) | 0.994 | 0.986 – 1.001 | 0.090 | **Albumin (g/L)** | 2.031 | 0.956 – 4.313 | 0.065 |
| Triglycerides (mg/dL) | 1.001 | 0.996 – 1.006 | 0.594 | **Portal hypertension-related bleeding** | 2.862 | 1.314 – 6.233 | 0.008 |
| Platelets (10^9^/L) | 1.003 | 0.996 – 1.008 | 0.373 | **MODEL 3** | | | |
| INR | 0.300 | 0.075 – 1.198 | 0.088 | **MELD-Na** | 0.937 | 0.853 – 0.953 | 0.005 |
| Albumin (g/L) | 2.049 | 1.081 – 3.885 | 0.028 | **Albumin (g/L)** | 2.627 | 1.136 – 6.074 | 0.024 |
| Bilirubin (mg/dL) | 0.723 | 0.504 – 1.038 | 0.078 | **Ascites** | 0.153 | 0.070 – 0.334 | <0.001 |
| Creatinine (mg/dL) | 0.372 | 0.187 – 0.740 | 0.005 | **MODEL 4** | | | |
| Child – Pugh score (points) | 0.610 | 0.465 – 0.802 | <0.001 | **Child – Pugh score (points)** | 0.685 | 0.518 – 0.904 | 0.008 |
| MELD-Na | 0.891 | 0.82 – 0.968 | 0.006 | **Creatinine (mg/dL)** | 0.274 | 0.099 – 0.763 | 0.013 |
| Multiple decompensation | 0.120 | 0.029 – 0.528 | 0.005 | **Total cholesterol (mg/dL)** | 0.988 | 0.979 – 0.996 | 0.008 |
| Ascites | 0.219 | 0.106 – 0.452 | <0.001 | **MODEL 5** | | | |
| Hepatic encephalopathy | 0.974 | 0.413 – 2.294 | 0.952 | **Child – Pugh score (points)** | 0.748 | 0.574 – 0.974 | 0.031 |
| Portal hypertension-related bleeding | 2.445 | 1.190 – 5.040 | 0.015 | **Creatinine (mg/dL)** | 0.414 | 0.199 – 0.857 | 0.018 |
| Statins | 1.399 | 0.687 – 2.846 | 0.354 | **Ascites** | 0.274 | 0.126 – 0.597 | 0.001 |
| Pioglitazone | 2.242 | 0.514 – 9.770 | 0.282 |  |  |  |  |
| GLP1r-a | 2.440 | 0.521 – 11.413 | 0.257 |  |  |  |  |
| SGLT2i | 0.676 | 0.083 – 5.529 | 0.715 |  |  |  |  |
| NSBBs | 0.771 | 0.342 – 1.740 | 0.532 |  |  |  |  |

BMI: Body Mass Index; INR: International Normalized Ratio; MELD: Model for end-stage liver disease; CAP; Controlled Attenuation Parameter; HVPG: Hepatic venous-portal gradient;

GLP1r-a: Glucagon-like peptide-1 receptor agonists; NSSBs: Non-selective β-blockers; SGLT2i: Sodium-glucose transport protein 2 inhibitors

*Adjusted BMI was calculated using a dry weight estimation calculated by subtracting a percentage of body weight based on fluid retention severity (5% for mild ascites, 10% for moderate, 15% for severe, and an additional 5% for bilateral pedal edema to the knees)

**Table S3. Predictors of extended hepatic recompensation (only considering Child Pugh B or C patients)**

|  | Univariable analysis | | |  | Multivariable analysis | | |
| --- | --- | --- | --- | --- | --- | --- | --- |
|  | **SHR** | **95% CI** | **p-value** |  | **aSHR** | **95% CI** | **p-value** |
|  |  |  |  | **MODEL 1** | | | |
| Age (years) | 0.998 | 0.969 – 1.028 | 0.926 | **Age (years)** | 0.979 | 0.944 – 1.016 | 0.279 |
| Sex. female | 2.073 | 0.025 – 4.191 | 0.142 | **MELD-Na** | 0.883 | 0.774 – 0.918 | 0.001 |
| Low-risk alcohol consumption | 0.689 | 0.299 – 1.588 | 0.383 | **Albumin (g/L)** | 1.936 | 1.043 – 3.593 | 0.036 |
| Adjusted BMI* (Kg/m^2^) | 0.992 | 0.927 – 1.061 | 0.822 | **Platelets (10^9^/L)** | 1.003 | 1.001 – 1.005 | 0.017 |
| Glycated hemoglobin (%) | 1.011 | 0.825 – 1.239 | 0.913 |  |  |  |  |
| Total cholesterol (mg/dL) | 1.007 | 0.999 – 1.015 | 0.076 |  |  |  |  |
| Triglycerides (mg/dL) | 1.004 | 0.996 – 1.013 | 0.283 |  |  |  |  |
| Platelets (10^9^/L) | 1.003 | 0.999 – 1.006 | 0.085 | **MODEL 2** | | | |
| INR | 0.161 | 0.041 – 0.623 | 0.008 | **MELD-Na** | 0.842 | 0.758 – 0.933 | 0.001 |
| Albumin (g/L) | 2.238 | 1.335 – 4.454 | 0.004 | **Albumin (g/L)** | 2.180 | 1.222 – 3.873 | 0.008 |
| Bilirubin (mg/dL) | 0.694 | 0.522 – 0.924 | 0.012 | **Platelets (10^9^/L)** | 1.002 | 1.001 – 1.004 | 0.035 |
| Creatinine (mg/dL) | 0.340 | 0.174 – 0.666 | 0.001 | **Multiple decompensation** | 0.151 | 0.030 – 0.772 | 0.023 |
| Child – Pugh score (points) | 0.618 | 0.433 – 0.880 | <0.001 |  |  |  |  |
| MELD-Na | 0.819 | 0.734 – 0.914 | <0.001 |  |  |  |  |
| Multiple decompensation | 0.166 | 0.038 – 0.722 | 0.017 |  |  |  |  |
| Ascites | 0.850 | 0.373 – 1.934 | 0.698 |  | | | |
| Hepatic encephalopathy | 0.669 | 0.286 – 1.564 | 0.354 |  |  |  |  |
| Portal hypertension-related bleeding | 0.529 | 0.131 – 2.137 | 0.372 |  |  |  |  |
| Statins | 0.656 | 0.313 – 1.377 | 0.266 |  |  |  |  |
| SGLT2i | 0.514 | 0.064 – 4.096 | 0.530 |  |  |  |  |
| NSBBs | 1.244 | 0.595 – 2.599 | 0.560 |  |  |  |  |

BMI: Body Mass Index; INR: International Normalized Ratio, MELD: Model for end-stage liver disease. FIB-4: Fibrosis-4; GLP1r-a: Glucagon-like peptide-1 receptor agonists; SGLT2i: Sodium-glucose transport protein 2 inhibitors, NSSBs: Non-selective β-blockers.

*Adjusted BMI was calculated using a dry weight estimation calculated by subtracting a percentage of body weight based on fluid retention severity (5% for mild ascites, 10% for moderate, 15% for severe, and an additional 5% for bilateral pedal edema to the knees)

**Table S4. Deaths and liver transplantation frequencies of the patients included according to recompensation**

| Patients | n = 124 patients | Extended recompensation  n = 52 | No recompensation  n = 72 | p value |
| --- | --- | --- | --- | --- |
| Deaths | 59 (48%) | 19 (37%) | 40 (56%) | 0.036 |
| - Liver-related deaths | 47 (38%) | 13 (25%) | 34 (47%) |  |
| - Liver failure | 36 (29%) | 10 (19%) | 26 (36%) |  |
| - Hepatocellular carcinoma | 11 (9%) | 3 (6%) | 8 (11%) |  |
| - Extrahepatic cancer | 1 (1%) | 1 (2%) | 0 |  |
| - Cardiovascular disease | 0 (0%) | - | - |  |
| - Others | 11 (9%) | 5 (10%) | 6 (8%) |  |
| Liver transplant (LT) | 18 (15%) | 6 (11%) | 12 (17%) | 0.424 |
| - Liver failure | 12 (10%) | 4 (8%) | 8 (11%) |  |
| - Hepatocellular carcinoma | 6 (5%) | 2 (4%) | 4 (6%) |  |

**Table S5. Deaths and liver transplantation frequencies according to recompensation stratified by Child Pugh score categories**

| Patients | Liver transplant (n=18) | p value | All-cause mortality  (n=59) | p value | Patient that eventually died (n=59) | Liver-related mortality  (n=47) | p value | Liver failure related mortality  (n=36) | p value |
| --- | --- | --- | --- | --- | --- | --- | --- | --- | --- |
| Child Pugh A (n=32) | 4 (22%) | | 17 (29%) | | **Child Pugh A (n=17)** | 12 (25%) | | 9 (25%) | |
| Recompensated (n=14) | 2 (11%) | 0.99 | 5 (8%) | 0.08 | **Recompensated (n= 5)** | 2 (4%) | 0.23 | 1 (3%) | 0.04 |
| Not recompensated (n=18) | 2 (11%) |  | 12 (20%) |  | **Not recompensated (n=12)** | 10 (21%) |  | 8 (22%) |  |
| Child Pugh B (n=76) | 10 (56%) | | 37 (63%) | | **Child Pugh B (n=37)** | 31 (66%) | | 23 (64%) | |
| Recompensated (n=16) | 3 (17%) | 0.99 | 3 (5%) | <0.01 | **Recompensated (n= 3)** | 2 (4%) | 0.843 | 1 (3%) | 0.63 |
| Not recompensated (n=50) | 7 (39%) |  | 34 (58%) |  | **Not recompensated (n=34)** | 29 (62%) |  | 22 (61%) |  |
| Child Pugh C (n=16) | 4 (22%) | | 5 (8%) | | **Child Pugh C (n=5)** | 4 (9%) | | 4 (11%) | |
| Recompensated (n=0) | 0 | - | 0 | - | **Recompensated (n= 0)** | 0 | - | 0 | - |
| Not recompensated (n=16) | 4 (22%) |  | 5 (8%) |  | **Not Recompensated (n= 5)** | 4 (9%) |  | 4 (11%) |  |

**Table S6. Deaths and liver transplantation frequencies according to recompensation stratified by MELD-Na score categories**

| Patients  (n=119) | Liver transplant (n=15) | p value | All-cause mortality  (n=57) | p value | Patients that eventually died (n=57) | Liver-related mortality  (n=45) | p value | Liver failure related mortality  (n=34) | p value |
| --- | --- | --- | --- | --- | --- | --- | --- | --- | --- |
| MELD-Na <10p (n=30) | 2 (13%) | | 14 (25%) | | **MELD-Na <10p (n=14)** | 13 (29%) | | 10 (29%) | |
| Recompensated (n=14) | 1 (7%) | 1.000 | 2 (4%) | 0.058 | **Recompensated (n=2)** | 1 (2%) | 0.14 | 0 | - |
| Not Recompensated (n=16) | 1 (7%) |  | 8 (14%) |  | **Not Recompensated (n=12)** | 12 (27%) |  | 10 (29%) |  |
| MELD-Na 10-15p (n= 57) | 7 (47%) | | 28 (49%) | | **MELD-Na 10-15p (n= 28)** | 19 (42%) | | 13 (38%) | |
| Recompensated (n=16) | 1 (7%) | 0.660 | 6 (11%) | 0.273 | **Recompensated (n=6)** | 3 (7%) | 0.34 | 2 (6%) | 0.77 |
| Not Recompensated (n=41) | 6 (40%) |  | 22 (39%) |  | **Not Recompensated (n=22)** | 16 (36%) |  | 9 (27%) |  |
| MELD-Na >15 (n=32) | 6 (40%) | | 15 (26%) | | **MELD-Na >15 (n=15)** | 13 (29%) | | 11 (32%) | |
| Recompensated (n=3) | 2 (13%) | 0.083 | 0 | - | **Recompensated (n=0)** | 0 | - | 0 | - |
| Not Recompensated (n=29) | 4 (27%) |  | 15 (26%) |  | **Not Recompensated (n=15)** | 13 (29%) |  | 11 (32%) |  |

*Death was classified as liver-related if it was a consequence of liver-failure or HCC. Liver failure-related death was considered if it occurred as a direct consequence of the progression of the underlying disease, whereas HCC-related death was defined as death resulting from complications directly attributable to HCC, including tumor progression or treatment failure.*

*P values correspond to the comparison between recompensated and non-recompensated patients for each clinical outcome (liver transplantation, all-cause mortality, liver-related mortality, and liver failure–related mortality) within each Child-Pugh and MELD-Na category.*

*Missing data of 5 patients regarding MELD-Na status*

**TABLE S7. Predictors of transplant-free survival considering recompensation as a non-time- dependent covariate**

|  | Univariable analysis | | | Multivariable analysis | | | |
| --- | --- | --- | --- | --- | --- | --- | --- |
| Patients | **HR** | **95%CI** | **p-value** |  | **aHR** | **95%IC** | **p-value** |
| Age (years) | 1.033 | 1.008 – 1.060 | 0.010 | **MODEL 1** | | | |
| Sex. male | 0.844 | 0.537 – 1.327 | 0.463 | **Age (years)** | 1.034 | 1.007 - 1.062 | 0.013 |
| Low-risk alcohol consumption | 0.789 | 0.469 – 1.329 | 0.373 | **Platelets** | 0.995 | 0.991 – 0.999 | 0.023 |
| Adjusted BMI (Kg/m^2^) (n=106) | 0.963 | 0.921 – 1.007 | 0.097 | **MELD-Na** | 1.065 | 1.015 – 1.117 | 0.010 |
| Glycated hemoglobin (%) | 1.0722 | 0.875 – 1.214 | 0.502 | **Recompensation** | 0.461 | 0.244 – 0.872 | 0.017 |
| Total cholesterol (mg/dL) | 0.998 | 0.992 – 1.005 | 0.628 |  |  |  |  |
| Triglycerides (mg/dL) | 0.996 | 0.991 – 1.002 | 0.213 |  |  |  |  |
| Bilirubin (mg/dL) | 1.205 | 1.092 – 1.329 | <0.001 |  |  |  |  |
| Albumin (g/L) | 0.727 | 0.517 – 1.021 | 0.066 |  | | | |
| INR | 1.097 | 0.694 – 1.732 | 0.693 |  |  |  |  |
| Platelets | 0.994 | 0.990 – 0.999 | 0.016 |  |  |  |  |
| Creatinine (mg/dL) | 1.387 | 1.128 – 1.705 | 0.002 |  |  |  |  |
| Child – Pugh score (points) | 1.216 | 1.046 – 1.415 | 0.011 |  |  |  |  |
| MELD- Na | 1.062 | 1.014 – 1.112 | 0.011 |  |  |  |  |
| FIB-4 | 1.004 | 0.989 – 1.019 | 0.589 |  |  |  |  |
| Recompensation | 0.330 | 0.174 – 0.628 | 0.001 |  |  |  |  |
| Extended recompensation | 0.279 | 0.168 – 0.465 | <0.001 |  |  |  |  |
| Statins | 1.381 | 0.865 – 2.206 | 0.176 |  |  |  |  |
| GLP1r-a | 1.476 | 0.463 – 4.704 | 0.511 |  |  |  |  |
| SGLT2i | 1.120 | 0.352 – 3.570 | 0.847 |  |  |  |  |
| NSBBs | 0.937 | 0.575 – 1.527 | 0.795 |  |  |  |  |

BMI: Body Mass Index; INR: International Normalized Ratio; MELD: Model for end-stage liver disease; CAP; Controlled Attenuation Parameter; HVPG: Hepatic venous-portal gradient;

GLP1r-a: Glucagon-like peptide-1 receptor agonists; NSSBs: Non-selective β-blockers; SGLT2i: Sodium-glucose transport protein 2 inhibitors

*Adjusted BMI was calculated using a dry weight estimation calculated by subtracting a percentage of body weight based on fluid retention severity (5% for mild ascites, 10% for moderate, 15% for severe, and an additional 5% for bilateral pedal edema to the knees)

**Table S8. Predictors of liver transplantation considering recompensation as a time-dependent covariate**

|  | Univariable analysis | | | Multivariable analysis | | | |
| --- | --- | --- | --- | --- | --- | --- | --- |
| Patients | **HR** | **95%CI** | **p-value** |  | **aSHR** | **95%IC** | **p-value** |
| Age (years) | 0.935 | 0.892 – 0.980 | 0.006 |  |  |  |  |
| Sex. male | 0.857 | 0.330 – 2.227 | 0.751 | **Age** | 0.940 | 0.889 – 0.993 | 0.027 |
| Adjusted BMI (Kg/m^2^) (n=106) | 0.950 | 0.852 – 1.050 | 0.360 | **Albumin** | 0.237 | 0.096 – 0.586 | 0.002 |
| Glycated hemoglobin (%) | 1.092 | 0.452 – 2.639 | 0.845 | **MELD-Na** | 1.191 | 1.091 – 1.301 | <0.001 |
| Total cholesterol (mg/dL) | 0.995 | 0.979 – 1.010 | 0.509 | **Recompensation** | 4.944 | 0.771 – 31.692 | 0.092 |
| Triglycerides (mg/dL) | 0.997 | 0.985 – 1.001 | 0.686 |  |  |  |  |
| Bilirubin (mg/dL) | 1.639 | 1.138 – 2.361 | 0.008 |  |  |  |  |
| Albumin (g/L) | 0.250 | 0.111 – 0.564 | 0.001 |  |  |  |  |
| INR | 2.818 | 1.507 – 5.267 | 0.001 |  |  |  |  |
| Platelets | 1.001 | 0.993 – 1.010 | 0.806 |  |  |  |  |
| Creatinine (mg/dL) | 1.303 | 1.014 – 1.674 | 0.039 |  |  |  |  |
| Child – Pugh score (points) | 2.208 | 1.530 – 3.190 | <0.001 |  |  |  |  |
| MELD- Na | 1.166 | 1.090 – 1.252 | <0.001 |  |  |  |  |
| FIB-4 | 1.024 | 0.999 – 1.051 | 0.059 |  |  |  |  |
| Recompensation | 0.619 | 0.140 – 2.746 | 0.528 |  |  |  |  |
| Extended recompensation | 0.267 | 0.060 – 1.185 | 0.083 |  |  |  |  |
| Statins | 0.560 | 0.161 – 1.951 | 0.363 |  |  |  |  |
| GLP1r-a | 0.782 | 0.103 – 5.922 | 0.812 |  |  |  |  |
| SGLT2i | 1.587 | 0.209 – 12.034 | 0.655 |  |  |  |  |
| NSBBs | 0.379 | 0.139 – 1.034 | 0.058 |  |  |  |  |

BMI: Body Mass Index; INR: International Normalized Ratio; MELD: Model for end-stage liver disease; CAP; Controlled Attenuation Parameter; HVPG: Hepatic venous-portal gradient;

GLP1r-a: Glucagon-like peptide-1 receptor agonists; NSSBs: Non-selective β-blockers; SGLT2i: Sodium-glucose transport protein 2 inhibitors

*Adjusted BMI was calculated using a dry weight estimation calculated by subtracting a percentage of body weight based on fluid retention severity (5% for mild ascites, 10% for moderate, 15% for severe, and an additional 5% for bilateral pedal edema to the knees)

**Figure S1. Cumulative incidence curves for extended recompensation and death/liver transplantation.**

**
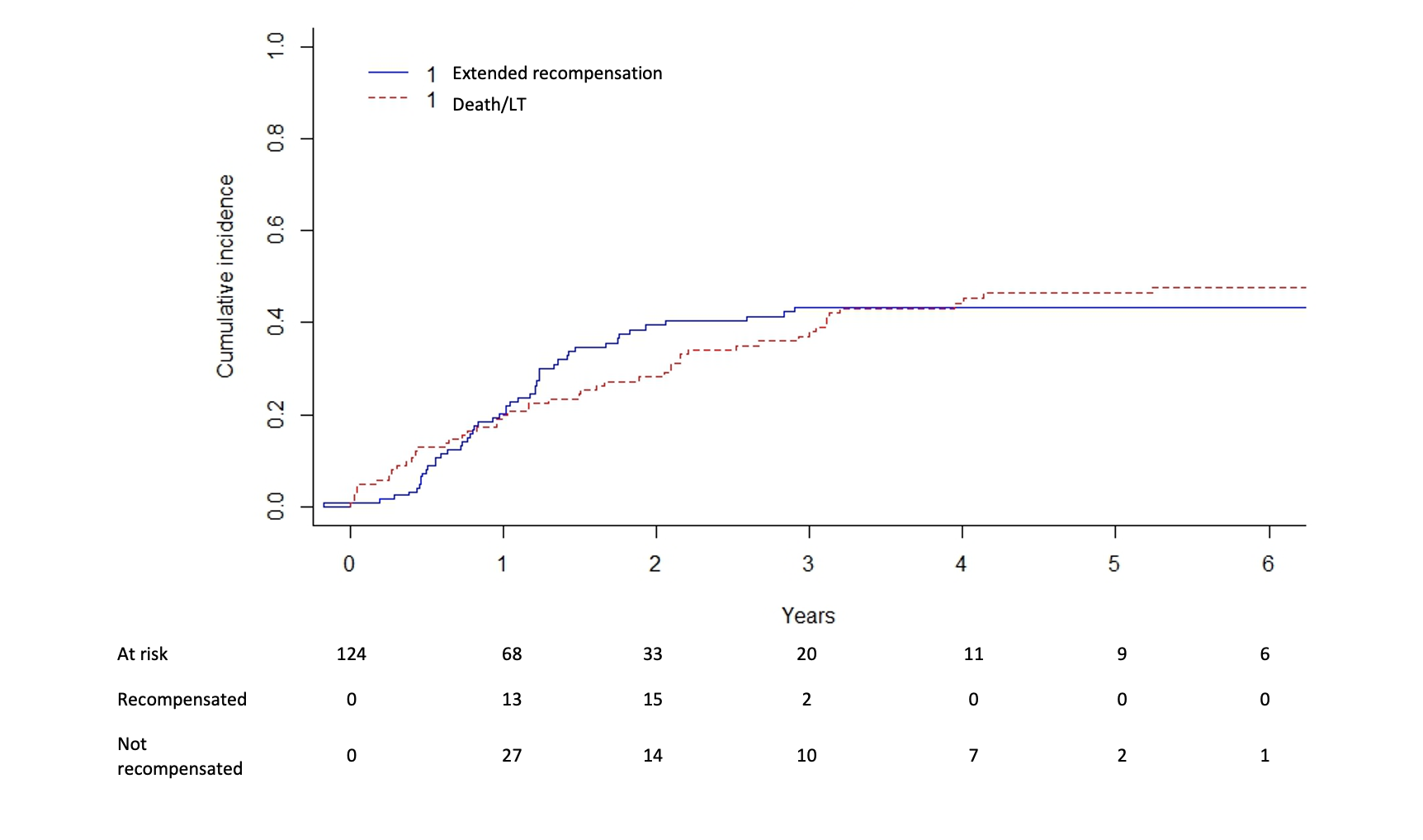
**

**Figure S2. Survival curves**

1. **Transplant free survival**

**
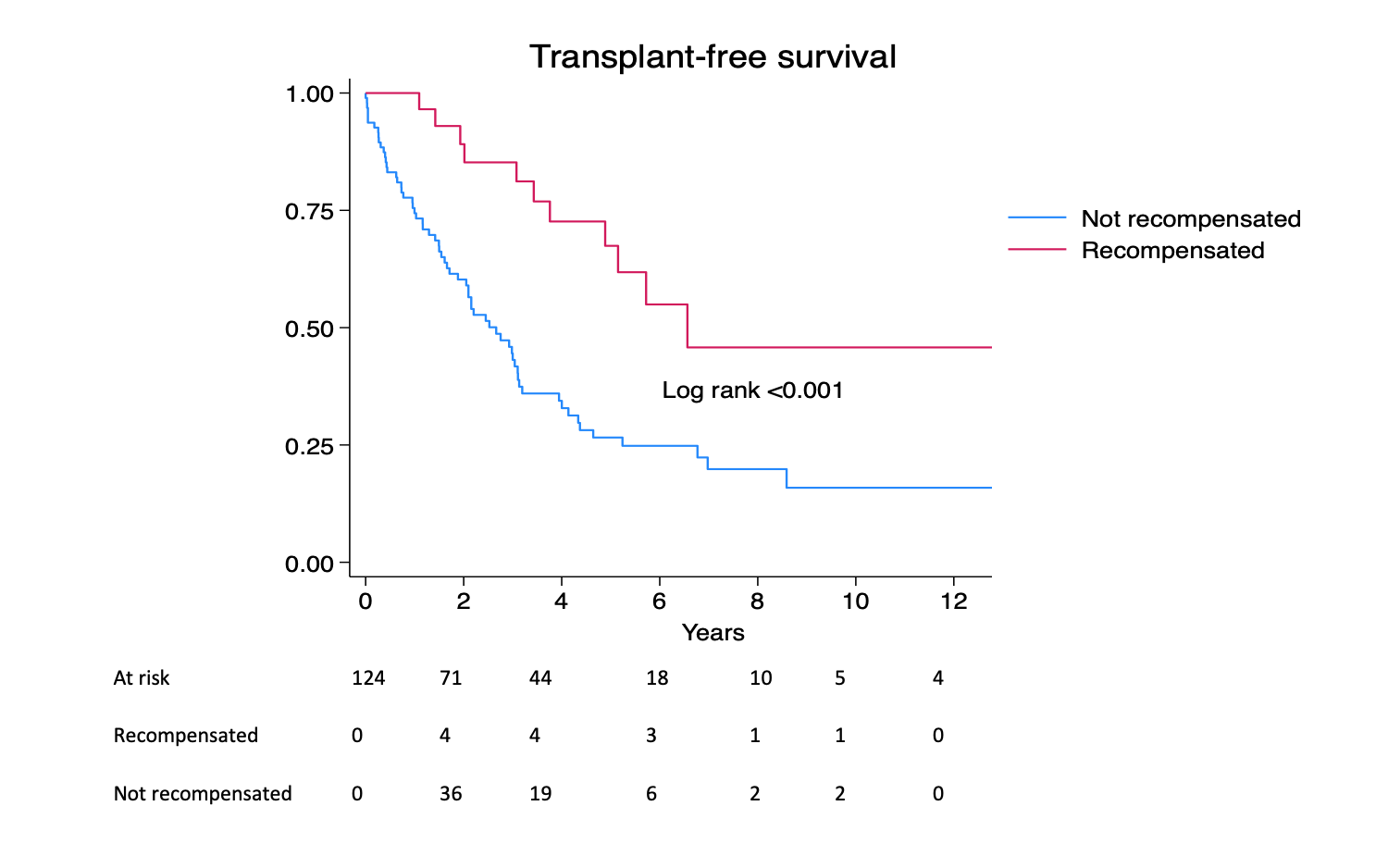
**

**b) Cumulative incidence for specific causes of death and liver transplant.**

**
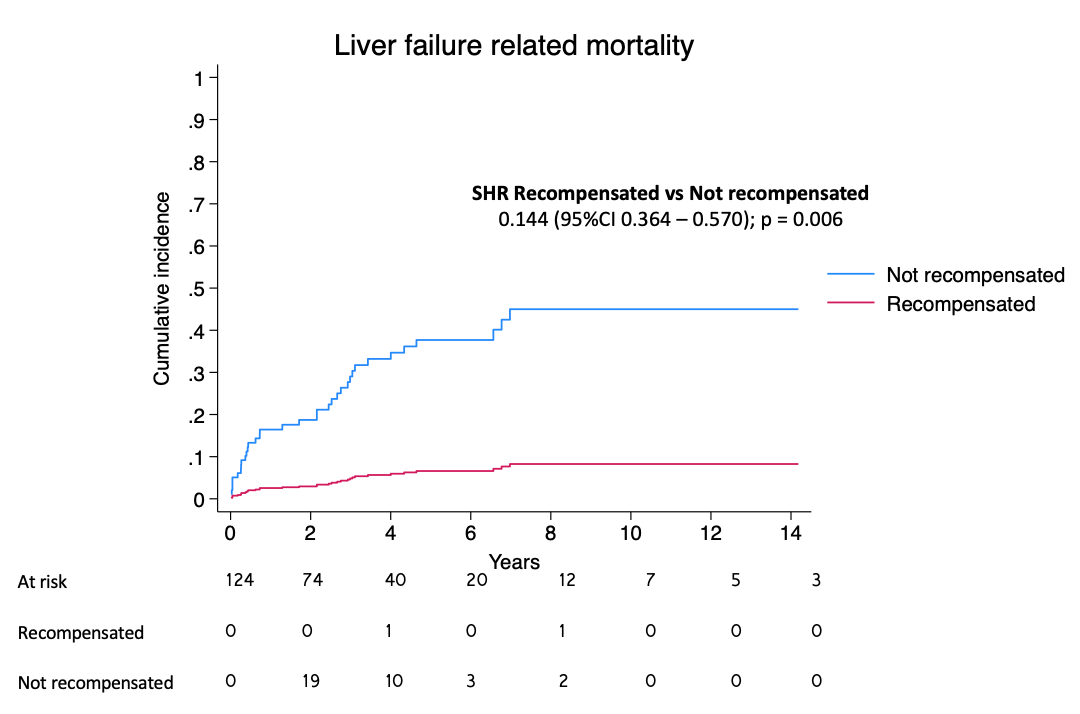
**

**
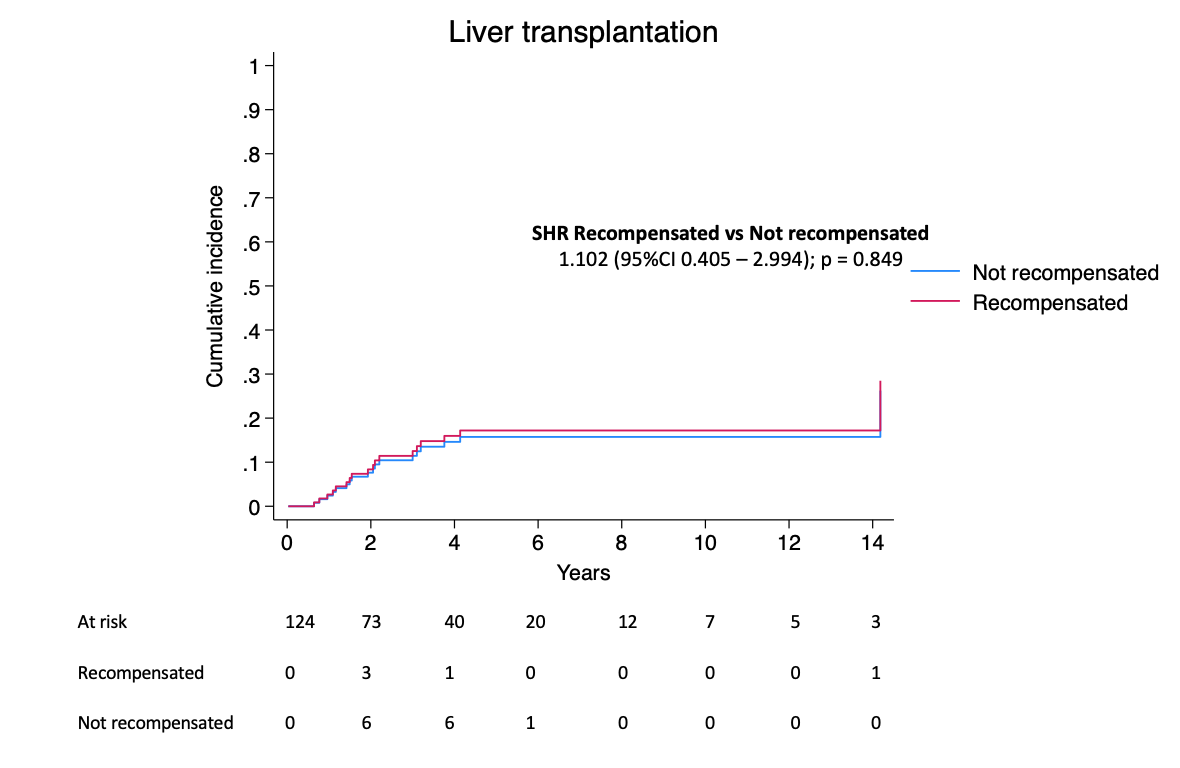
**

**
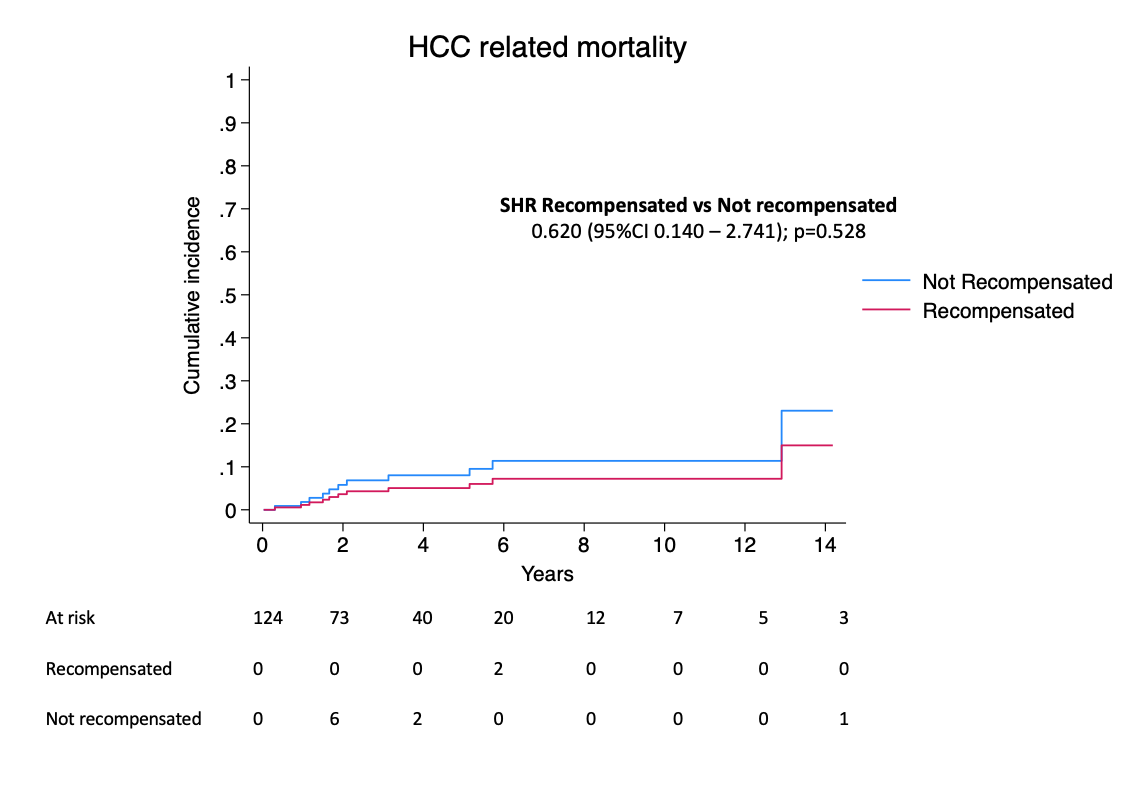
**

**
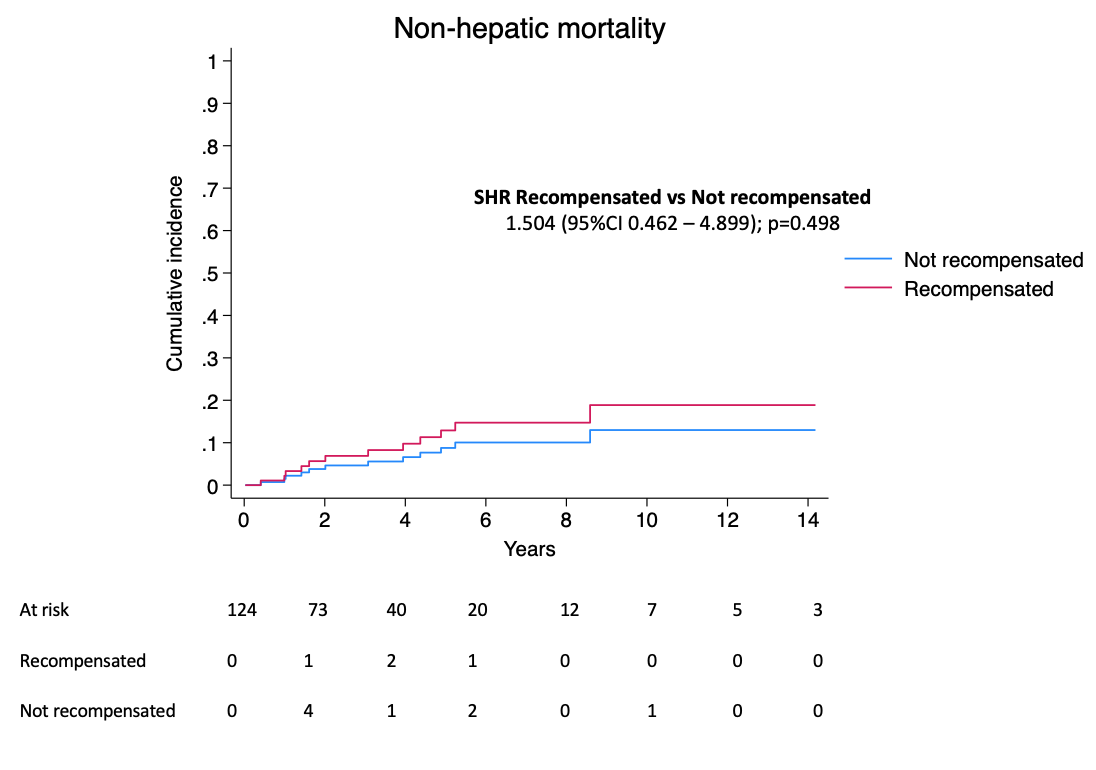
**
